# Retraction Note: MicroRNA-143 targets MAPK3 to regulate the proliferation and bone metastasis of human breast cancer cells

**DOI:** 10.1186/s13568-024-01814-0

**Published:** 2024-12-27

**Authors:** Yiqun Du, Jian Zhang, Yanchun Meng, Mingzhu Huang, Wangjun Yan, Zhiqiang Wu

**Affiliations:** 1 Department of Oncology, Shanghai Medical College, Fudan University, Shanghai, 200032 China; 2 Department of Medical Oncology, Fudan University Shanghai Cancer Center, Shanghai, 200032 China; 3 Department of Musculoskeletal Oncology, Fudan University Shanghai Cancer Center, 270 Dong’an Road, Shanghai, 200032 China

**Retraction Note to: Du et al. AMB Expr (2020) 10:134** 10.1186/s13568-020-01072-w

Editor-in-Chief has retracted this article at the request of the corresponding author. Following publication, concerns were raised about similarities between the published figures and those in previously published articles. Specifically:

- Figure 2C appears to have partial overlap with Figure 2 in (Yu et al. 2018);
- Figure 2D appears to have partial overlap with Figure 1E in (Chen et al. 2019) and Figure 4 in (Li et al. 2018);
- Figures 3A, B appear to have partial overlap with Figure 3D in (Qiu et al. 2018); and
- Figures 3C, D appear to have partial overlap with Figure 4 in (Ningning et al. 2019) when flipped.

The corresponding author has stated that the affected figures were obtained through a commercial lab hired to perform some of the experiments for this study.

The Editor-in-Chief therefore no longer has confidence in the integrity of the data in this article.

Yiqun Du and Zhiqiang Wu agree with this retraction, but not the wording of this retraction notice. None of the other authors have responded to any correspondence from the editor or publisher about this retraction.
